# Breath-Holding Episodes in Riboflavin Transporter Deficiency Type 2: A Case Report of Diagnostic Challenges and Successful Riboflavin Treatment

**DOI:** 10.7759/cureus.119691

**Published:** 2026-11-15

**Authors:** Abigail Fox, Irina Luca

**Affiliations:** 1 General Paediatrics, St George's Hospital, London, GBR

**Keywords:** breath-holding, brown-vialetto-van laere syndrome, pontobulbar palsy, riboflavin, riboflavin transport deficiency, sensorineural (sn) hearing loss

## Abstract

Riboflavin transporter deficiency (RTD), previously known as Brown-Vialetto-Van Laere syndrome, is a rare autosomal recessive neurodegenerative disorder characterised by pontobulbar palsy, sensorineural hearing loss, and respiratory compromise. The condition is caused by pathogenic variants in the riboflavin transporter genes *SLC52A2* and *SLC52A3*, which encode the human riboflavin transporters RFVT2 and RFVT3, respectively. Treatment with high-dose riboflavin has been shown to improve clinical outcomes. Several cases have been reported, with considerable variation in age at onset, disease progression, and clinical outcomes.

We describe the case of a 19-month-old girl who presented with significant breath-holding episodes, progressive pontobulbar palsy, sensorineural hearing loss, and global developmental delay. Whole-genome sequencing identified a novel homozygous missense variant in *SLC52A2*, confirming a diagnosis of RTD type 2. The patient required long-term oxygen therapy and percutaneous gastrostomy to support respiration and feeding, respectively. Consistent with findings in other reported cases of RTD, the patient responded favourably to high-dose riboflavin treatment, demonstrating improvements in motor function, although complete neurological recovery was not achieved.

RTD should be considered in patients presenting with pontobulbar palsy, sensorineural hearing loss, and respiratory compromise. Breath-holding episodes and apnoeas may provide early clinical clues to the diagnosis. Early recognition is crucial because treatment with high-dose riboflavin may improve clinical outcomes and the potential for neurological recovery.

## Introduction

Riboflavin transporter deficiency (RTD), previously known as Brown-Vialetto-Van Laere syndrome, is a rare neurodegenerative disorder caused by pathogenic variants in the riboflavin transporter genes *SLC52A2 *and *SLC52A3*. RTD is characterised by progressive pontobulbar palsy, sensorineural hearing loss, muscle weakness and respiratory compromise ​[1,2]. RTD type 2 (RTD2), caused by pathogenic variants in *SLC52A2*, is more commonly associated with sensory ataxia, optic nerve involvement, and predominant upper limb weakness, whereas RTD type 3 (RTD3), caused by pathogenic variants in *SLC52A3*, is more frequently associated with facial weakness and respiratory involvement ​[3]. Nevertheless, considerable phenotypic overlap exists between the two subtypes, and definitive diagnosis relies on molecular genetic testing. If left untreated, RTD is associated with significant morbidity and mortality, with respiratory failure accounting for most reported deaths ​[2]. More than 100 cases have been reported in the literature, with considerable variation in age at onset, disease progression and clinical outcomes.

Riboflavin is the precursor of the flavin cofactors flavin mononucleotide (FMN) and flavin adenine dinucleotide (FAD), which are essential for mitochondrial energy production and fatty acid metabolism ​[4]. Three human riboflavin transporters, RFTV1-3, which facilitate the transport of dietary riboflavin across cell membranes, have been identified. These transporters are encoded by *SLC52A1-3*, respectively, and are expressed in a range of tissues. RFVT2 is abundant in nervous tissue, whereas RFVT3 is expressed in the intestine, testes and prostate. In RTD, pathogenic variants in *SLC52A2* and *SLC52A3* impair cellular riboflavin uptake, resulting in reduced availability of FMN and FAD. Although the mechanisms underlying the clinical manifestations of RTD are not fully understood, current evidence suggests that oxidative stress, mitochondrial dysfunction and dysregulated energy metabolism play important roles ​[5,6].

High-dose riboflavin treatment has been shown to be life-saving in patients with RTD. In a recent review of published cases, 80.9% of patients showed overall clinical improvement following riboflavin treatment, while the remaining 19.1% remained clinically stable ​[7]. No deaths were reported among patients who received riboflavin treatment. Studies have shown that clinical outcomes improve when riboflavin treatment is initiated early in the disease course ​[8,9]. It is hypothesised that prolonged periods without treatment may lead to irreversible damage to motor and sensory neurons ​[9].

We report the case of a 19-month-old child with RTD2 who presented with pathological respiratory episodes characterised by breath-holding and profound apnoeas. It is important to distinguish these events from the common benign breath-holding spells observed in young children, which are reflex autonomic events typically precipitated by emotional stimuli, pain or frustration. Such spells are generally brief and self-limiting, with spontaneous recovery, and occur in children with otherwise normal neurological development and examination ​[10]. In contrast, respiratory episodes associated with RTD may occur spontaneously, without an identifiable trigger, and can progressively increase in frequency and severity. Rather than representing transient autonomic reflexes, these episodes may reflect underlying cranial neuropathy and motor neuropathy affecting the respiratory muscles.

Breath-holding episodes are not frequently reported in the RTD2 literature. A case report from China identified breath-holding episodes as a presenting feature of RTD2 ​[11]. Furthermore, a separate study described four children who experienced breath-holding episodes within the first year following presentation ​[12]. Although the literature remains limited, pathological breath-holding episodes may form part of the clinical spectrum of RTD2 and potentially provide an early diagnostic clue.

This case highlights the importance of considering RTD in children presenting with unexplained or progressive respiratory episodes, particularly when these occur alongside sensorineural hearing loss and pontobulbar palsy. It also reinforces the potential for significant clinical improvement with early riboflavin therapy.

## Case presentation

Patient information

We describe a 19-month-old girl born to healthy, consanguineous Pakistani parents following an uneventful pregnancy and a full-term spontaneous vaginal delivery. She had an uneventful neonatal period and passed her newborn hearing screen. An innocent murmur was identified at two months of age. Gross motor and speech milestones were delayed. She rolled at nine months and crawled at 15 months. At the time of admission (19 months), she was able to pull to stand but was unable to stand or walk independently. At presentation, she was largely non-verbal, able to babble but unable to produce any comprehensible words. She had retained some receptive language skills and used gestures and pointing to communicate her needs.

She presented with significant breath-holding episodes that began at six months of age and were initially thought to be benign. Other children in the extended family had experienced similar breath-holding episodes from which they had subsequently recovered, contributing to the delayed presentation in this case. The episodes of breath-holding became progressively more frequent and prolonged. Initially, they responded well to stimulation; however, a prolonged episode of apnoea resulted in a respiratory arrest in hospital, requiring chest compressions and manual ventilation. Once respiratory effort was re-established, the child was stabilised on high-flow nasal cannula oxygen therapy.

She was also noted to have swallowing difficulties and frequent choking with both solids and liquids. She had previously attended the accident and emergency department on multiple occasions with lower respiratory tract infections requiring antibiotics, which were retrospectively attributed to aspiration events.

A chronological overview of the patient's clinical presentation, investigations, therapeutic interventions and outcomes is provided in Figure 1.

**Figure 1:**
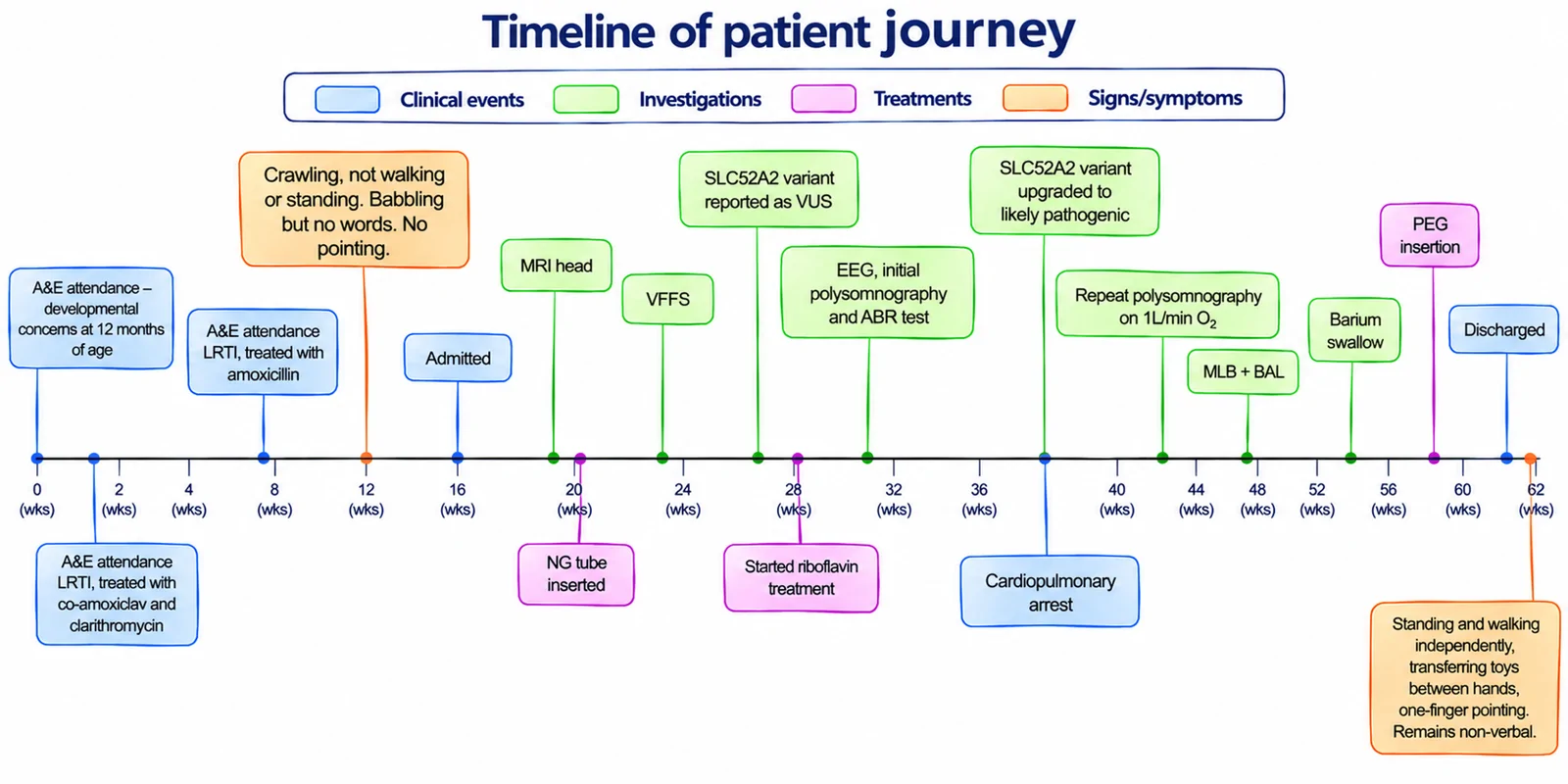
Chronological timeline of key clinical milestones in the patient's journey. The timeline spans from first hospital attendance at 12 months of age (week 0) to post-discharge from hospital (week 62). The timeline shows clinical events (blue), investigations (green), treatments (pink) and signs/symptoms (orange). A&E: accident and emergency; LRTI: lower respiratory tract infection; MRI: magnetic resonance imaging; NG: nasogastric; VFFS: videofluoroscopic swallow study; VUS: variant of uncertain significance; EEG: electroencephalogram; ABR: auditory brainstem response; MLB: microlaryngoscopy and bronchoscopy; BAL: bronchoalveolar lavage; PEG: percutaneous endoscopic gastrostomy

Clinical findings

Examination revealed an underweight child with normal vital signs and no respiratory distress at rest. She had symmetrical facial features without dysmorphism. Neurological examination was difficult to complete because of the severity of her breath-holding episodes. Attempts at examination frequently triggered distress, resulting in loss of consciousness, profound oxygen desaturation, and cyanosis. Observational assessment revealed both central and peripheral hypotonia, as well as an inability to stand or walk independently at 19 months of age. She exhibited bilateral vertical and horizontal nystagmus, with no evidence of ptosis. Ophthalmological examination revealed light sensitivity, difficulty tracking objects and reduced visual alertness. Swallow assessment identified frequent choking with both solids and liquids, accompanied by oxygen desaturation. The gag reflex was preserved, with appropriate movement of the lips and tongue. The child also demonstrated reduced responses to loud sounds and did not respond to her name.

Diagnostic assessment

Given the age at presentation and the predominance of breath-holding episodes, benign breath-holding spells were considered as a possible diagnosis. As symptoms progressed, other differential diagnoses, including epilepsy, structural brain disorders, metabolic disorders and Rett syndrome, were considered.

The patient’s full blood count revealed normocytic, hypochromic anaemia, which was confirmed by blood film examination. Serum iron was low at 5 µmol/L (reference range: 14-30 µmol/L), while ferritin, folate, and vitamin B12 levels were normal. Basic biochemistry results were unremarkable. A metabolic panel, including ammonia, lactate, serum amino acid profile, blood spot acylcarnitine analysis and urinary organic acids, was unremarkable. A full acylcarnitine profile was unavailable. Blood riboflavin levels were normal at 399 nmol/L (reference range: 175-470 nmol/L).

Microlaryngoscopy and bronchoscopy demonstrated normal airway anatomy, although a cobblestone appearance of the mucosa was noted, suggesting reflux. This was further investigated using a barium swallow and a videofluoroscopic swallow study, which demonstrated significant oropharyngeal dysphagia, with frequent aspiration across all consistencies.

Initial polysomnography demonstrated significant sleep-disordered breathing, with an elevated apnoea-hypopnoea index (AHI) of 12.4 events/hour and an oxygen desaturation index (ODI) of 15.3 events/hour, based on oxygen desaturations of at least 3% from baseline. Snoring was recorded during 27% of total sleep time, suggesting a component of upper airway obstruction. In addition, central apnoeic events occurred at an average frequency of three events/hour, indicating an abnormal central respiratory pattern. In view of these findings, the child was commenced on supplemental oxygen at 1 L/min overnight to support nocturnal respiratory function.

Magnetic resonance imaging (MRI) of the brain demonstrated structurally normal brain parenchyma, although the pons was noted to be slender in appearance, a non-specific finding in the context of developmental abnormalities (Figure 2). Electroencephalography (EEG) showed no evidence of epileptiform activity. Auditory brainstem response (ABR) testing and behavioural audiometry revealed bilateral auditory neuropathy spectrum disorder (ANSD). Electromyography (EMG) could not be performed due to the severity of the child's breath-holding episodes, representing a limitation in the neurological assessment.

**Figure 2:**
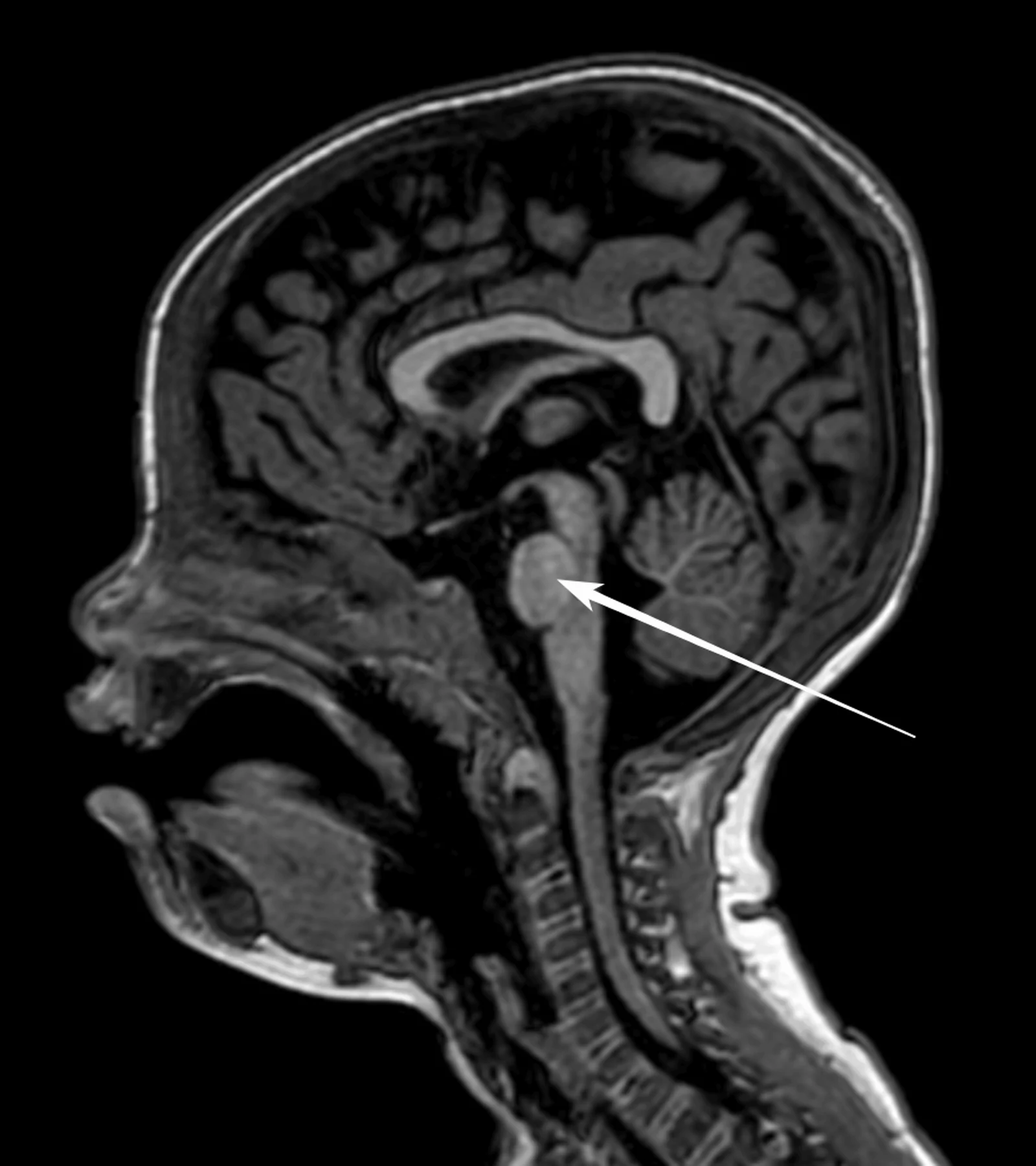
Slender pons on brain MRI. Sagittal T1-weighted brain MRI image acquired at 19 months of age. The image demonstrates structurally normal brain parenchyma with a slender pons, and non-specific finding in the context of developmental abnormalities, shown by the white arrow.

The diagnosis was ultimately confirmed by rapid whole-genome sequencing, which identified a homozygous missense variant in the *SLC52A2* gene (NM_024531.5:c.332C>A; p.(Ser111Tyr)). Both parents were confirmed to be heterozygous for the variant. Targeted testing for the *SLC52A2* variant confirmed that a younger sibling was unaffected. The variant was initially classified as a variant of uncertain significance (VUS) using the ACMG/AMP and ACGS 2024 guidelines. Following further assessment, the classification was upgraded to likely pathogenic. This reclassification was based on additional evidence from in-house in silico protein modelling that suggests a deleterious effect of p.(Ser111Tyr) by severely destabilising the *SLC52A2* transporter (PM1_supporting). Furthermore, the patient's characteristic clinical features of RTD provided additional supporting evidence (PP4_supporting). Together with the previously established evidence of rarity in the population database (PM2_moderate), homozygosity in the patient (PM3_supporting) and predicted deleterious effect on protein function (PP3_supporting), these findings supported reclassification as likely pathogenic.

Therapeutic intervention

The patient was commenced on oral riboflavin at a dose of 50 mg/kg/day, with no identifiable adverse effects.

The child experienced recurrent respiratory tract infections and persistent chest radiographic changes secondary to aspiration, necessitating antibiotic treatment. Bronchoalveolar lavage demonstrated heavy growth of *Pseudomonas aeruginosa* and *Staphylococcus aureus*, for which prolonged courses of ciprofloxacin and flucloxacillin were administered. Long-term supplemental oxygen was required overnight to manage respiratory compromise. Due to an unsafe swallow, the child initially received enteral nutrition via a nasogastric tube. A percutaneous gastrostomy was subsequently inserted to reduce aspiration risk and facilitate long-term nutritional support.

The child also had persistent normocytic, hypochromic anaemia, which responded poorly to high-dose ferrous fumarate and intravenous iron infusions. Vitamin C and a multivitamin supplement were commenced to support iron absorption and nutritional status. Throughout the prolonged admission, the child received multidisciplinary support from physiotherapy, occupational therapy, and speech and language therapy. Regular input from play therapists supported developmental progress.

Follow-up and outcomes

After three months of riboflavin treatment, polysomnography demonstrated a marked improvement in sleep-disordered breathing, with the AHI reduced to 1.0 events/hour and the ODI to 0.0 events/hour. Snoring was also reduced, occurring during only 5.1% of total sleep time, with no central apnoeas recorded. Notably, this study was performed with the patient receiving their baseline supplemental oxygen of 1 L/min.

After six months of riboflavin treatment, the child’s gross motor development had improved significantly. She was able to stand and walk independently, transfer toys between hands and engage in play. However, she remained non-verbal due to profound sensorineural hearing loss, although she was making progress in using Makaton with support from speech and language therapists. Bilateral cochlear implantation was planned for the future. The child’s swallow remained unsafe, and she continued to receive no oral intake, with all feeds and medications administered via gastrostomy.

The child was discharged home following a prolonged hospital admission, with community support from social services, nursing, physiotherapy and speech and language teams. She continued to receive follow-up care from the metabolic, respiratory, community paediatric, ophthalmology and audiology teams.

## Discussion

This case demonstrates several characteristic features of *SLC52A2*-related disease, including early-onset hypotonia, developmental delay, sensorineural hearing loss, and respiratory compromise. The child was unable to stand or walk independently at 19 months of age and had a history of recurrent respiratory tract infections secondary to aspiration, suggestive of significant bulbar dysfunction. The combination of these symptoms should raise suspicion for RTD, particularly in a child with otherwise unexplained neurological deterioration. In published reviews of molecularly confirmed RTD cases, motor neuropathy and sensorineural hearing loss were among the most prominent manifestations, while respiratory compromise and bulbar palsy were also frequently reported ​[3,4].

A particularly notable feature of this case was the severity of the child's breath-holding episodes and apnoeas. These episodes made neurological examination challenging, meaning that initial assessment relied heavily on clinical observation. It is important to distinguish the observed breath-holding episodes from central apnoea, as these may represent different underlying physiological mechanisms. In our patient, central apnoeic events were documented objectively on polysomnography. However, the precise relationship between the observed breath-holding episodes and central apnoeas remains uncertain. This unusual respiratory presentation may have contributed to delays in the investigation and diagnosis of this rare disorder. Breath-holding has previously been reported in association with *SLC52A2*-related disease, although it appears to be an uncommon presenting feature. A previous case report described a child with RTD2 who developed frequent cyanotic breath-holding spells alongside upper limb hypotonia and pontobulbar dysfunction, all of which improved following riboflavin treatment ​[11]. The prominent breath-holding and apnoeic episodes in our patient may therefore represent an additional clinical clue to RTD.

Our patient’s clinical response to riboflavin treatment was encouraging. Following three months of riboflavin treatment, repeat polysomnography demonstrated complete resolution of the previously documented central apnoeic events, with the average frequency decreasing from 3.0 events/hour before treatment to 0.0 events/hour. Her hypotonia also improved, accompanied by meaningful gains in functional abilities. Whereas she was unable to stand or walk independently at 19 months of age, she subsequently acquired these abilities following riboflavin treatment. Improvements in fine motor skills were also observed, including transferring toys between hands, using single-finger pointing to indicate needs and engaging in play. This degree of neurological recovery is consistent with previous reports demonstrating improvement in motor, pontobulbar and respiratory function ​[2,3,7]. Nevertheless, the contribution of concurrent multidisciplinary therapies should be acknowledged, as these may have contributed to her developmental progress.

Despite the marked improvement in motor and respiratory function, no improvement was seen in the child’s sensorineural hearing loss within six months of treatment. This finding is consistent with published reports that demonstrate that audiological response to riboflavin is variable, in contrast to more generalised improvement in other neurological fields ​[13-15]. A recent longitudinal case study of 11 children with RTD found that improvements in hearing are predominantly confined to the first 12 months of riboflavin treatment, with better outcomes in children treated earlier in the disease course ​[9]. The author suggests that a prolonged period between disease onset and treatment may result in irreversible neuronal injury, which further supports the importance of early diagnosis and initiation of riboflavin treatment in RTD.

Diagnosis was established via whole-genome sequencing, which identified a homozygous missense variant in *SLC52A2*. The phenotype of RTD can overlap with numerous neuromuscular and neurodegenerative disorders. Therefore, comprehensive genetic testing can be crucial in achieving a timely diagnosis, particularly when symptoms are severe and rapidly evolving. The identification of a novel variant further contributes to the expanding genotype of RTD2.

## Conclusions

This case highlights the broad clinical spectrum of RTD and the importance of considering this disorder in children presenting with a combination of motor neuropathy, sensorineural hearing loss, developmental delay and respiratory compromise. Progressive breath-holding episodes or apnoeas may represent additional diagnostic clues, although they are not specific to RTD. Early genetic diagnosis is essential to facilitate timely initiation of riboflavin treatment and multidisciplinary management, which may improve clinical outcomes. However, the observed clinical improvement in this case should be interpreted cautiously, as the contribution of riboflavin treatment cannot be entirely distinguished from that of concurrent supportive and rehabilitative interventions. Furthermore, neurological recovery may vary across clinical domains, with established neurological deficits potentially demonstrating limited responsiveness to treatment. Given the single-case nature of this report, these observations should be regarded as supportive of the potential benefits of early recognition and treatment rather than definitive evidence of treatment efficacy.
